# How can the use of data within the immunisation programme be increased in order to improve data quality and ensure greater accountability in the health system? A protocol for implementation science study

**DOI:** 10.1186/s12961-018-0312-2

**Published:** 2018-05-03

**Authors:** Binyam Tilahun, Alemayehu Teklu, Arielle Mancuso, Zeleke Abebaw, Kassahun Dessie, Desalegn Zegeye

**Affiliations:** 1 http://www.eHealthLab.org; 2 Department of Pediatrics, School of Medicine, University of Gondar, Gondar, Ethiopia; 3 Policy and Planning Directorate, Ministry of Health, Addis Ababa, Ethiopia; 4 Alliance for Health Policy and Systems Research, World Health Organization, Geneva, Switzerland

**Keywords:** Immunisation, Implementation science, Data use, Data quality, Accountability

## Abstract

**Background:**

Immunisation remains one of the most important and cost-effective interventions to reduce vaccine-preventable child morbidity, disability and mortality. Health programmes like the Expanded Program of Immunization rely on complex decision-making and strong local level evidence is important to effectively and efficiently utilise limited resources. Lack of data use for decision-making at each level of the health system remains the main challenge in most developing countries. While there is much evidence on data quality and how to improve it, there is a lack of sufficient evidence on why the use of data for decision-making at each level of the health system is low. Herein, we describe a comprehensive implementation science study that will be conducted to identify organisational, technical and individual level factors affecting local data use at each level of the Ethiopian health system.

**Methods:**

We will apply a mixed methods approach using key informant interviews and document reviews. The qualitative data will be gathered through key informant interviews using a semi-structured guide with open- and closed-ended questions with four categories of respondents, namely decision-makers, data producers, data users and community representatives at the federal, regional, zonal, *woreda* and community levels of the health system. The document review will be conducted on selected reports and feedback documented at different levels of the health system. Data will be collected from July 2017 to March 2018. Descriptive statistics will be analysed for the quantitative study using SPSS version 20 software and thematic content analysis will be performed for the qualitative part using NVivo software.

**Discussion:**

Appropriate and timely use of health and health-related information for decision-making is an essential element in the process of transforming the health sector. The findings of the study will inform stakeholders at different levels on the institutionalisation of evidence-based practice in immunisation programmes.

## Background

WHO has estimated that 29% of under-five deaths could be prevented with existing vaccines, averting between 2 and 3 million deaths each year globally [1]. Worldwide immunisation coverage showed improvement in the past years; however, the validity of the data for measuring change over time has been questioned [2]. Therefore, accurate immunisation information is essential for decision-makers of the Expanded Program on Immunization (EPI) to track and improve programme performance [3].

Over the past two decades, the government of Ethiopia has invested heavily in health system strengthening, which helped the country remarkably achieve most of the Millennium Development Goal targets. Among the notable achievements, Millennium Development Goal 4 was achieved with a 67% drop in under-five mortality from the 1990 estimate, contributing to an increase in average life expectancy at birth from 45 years in 1990 to 64 years in 2014 [4]. Currently, under the Sustainable Development Goals call to action, Ethiopia agreed to end preventable deaths of newborns and children with the aim to reduce under-five mortality to at least as low as 25 per 1000 live births by 2030 [5].

Ethiopia has a decentralised, three-tier system comprising primary, secondary and tertiary levels of care. The primary healthcare unit, through the health extension programme, which is an innovative community based strategy to deliver preventive and promotive services at community level, is the backbone of the routine immunisation programme [6, 7]. According to the Ethiopian Demographic and Health Survey 2016 report [8], complete immunisation coverage was 38.5% at the national level and 45.8% in the Amhara region. Further, this data generally showed vaccination coverage to be lower than that obtained from the routine service statistics of the Ministry of Health, raising questions of data quality and reporting barriers in the health system. Further, a comparative analysis performed by USAID on immunisation data indicated a 12% disparity in complete vaccination coverage between routine Health Management Information System and survey coverage data, showing data quality problems [9]. Programme data showed the presence of fabricated reports in some facilities due to incentive needs. At district level, the most common challenge was the reporting of data to the next level without or with minimal use or processing [8, 10, 11], with the main implementation barriers being individual and technical level constraints [9].

Ensuring well-coordinated activities to foster high immunisation coverage is dependent on the availability of timely, accurate and complete information pertaining to vaccinations. Thus, the need for multidimensional, accurate and timely information is high in order to address issues related to quality and equity in the health sector. Our argument is that the quality of data and, consequently, that of the information system must be assessed with a broader perspective, focusing on support mechanisms [12, 13] as well as on technicalities (data collection tools and the reporting system).

With this in mind and under the health sector transformation plan, Ethiopia set an ‘information revolution’ as a priority agenda to bring fundamental cultural and attitudinal change regarding the perceived value and practical use of information. However, the prevailing practice in terms of effectively utilising information remains unsatisfactory and the quality of information an unsolved problem in the health sector [14]. Additionally, there is also weak use of data, mostly attributed to the high degrees of fragmentation across multiple parallel information subsystems, a lack of community engagement and severely constrained information system infrastructure and human resources. With the availability of complete and accurate data, data use for evidence-informed decisions could improve data quality [14]. However, the practical utility of health information, as well as how often and how effectively data is used or not, is determined by multiple factors, which can be categorised into three general categories, namely the attitudes and actions of people who produce or use data, the technical aspects of data processes and tools, and the organisational context that supports data processes [15].

Recent studies reported inconsistencies in data reporting as well as poor support mechanisms to ensure data quality at the district level [16]. For example, a study in Nepal found that lower volumes of data were obtained from the facility registers compared to data volumes reported at the district level [17], showing a tendency of over-reporting at higher levels. Other studies showed that errors in reporting were due to a lack of supervision and feedback from the superior levels as well as inadequate incentives to health workers [18, 19]. Further, a study from Uganda showed that there was low information use (24%), which was consistent with the observed limited skills level to interpret (41%) and use information (44%) [20].

A study on the health information system in Ethiopia has shown that data management and use for decision-making were not adequate at lower health system levels, and that data quality assurance and feedback mechanisms were weak [21, 22]. Another study on data verification also revealed that there were incomplete and poor-quality reports across different programmes, compromising decisions and allocation of already scarce resources [11]. A data quality and information use assessment in Ethiopia showed a limited culture of using information for decision-making, where only 37% of the facilities exercised discussion and made decisions using findings from routine health information [12]. Similarly, there was also inadequate supervision and feedback from senior levels to address problems of inadequate documentation, late and incomplete reporting, and inaccurate reporting [12]. In Ethiopia, there have been significant recent investments in establishing and expanding information systems in recognition of the significant role data availability and use play in improving health service delivery. While some of these systems have contributed to the strides made in Ethiopia’s health sector, multiple systems remain fragmented, with highly variable data quality and uneven implementation [23].

The effective use of the data flowing through these systems has not been institutionalised at all levels of the health system and the Federal Ministry of Health’s ability to direct the development of improved functionality is limited. Good health information systems are crucial for addressing health challenges and improving health service delivery in developing countries. In addition, the value of health information is determined by its utilisation in decision-making. However, the quality of the data produced by such systems is often poor and the data are not used effectively for decision-making [24]. In Ethiopia, data quality and utilisation of health information remains weak, particularly at primary healthcare facilities and district levels [25].

The efficient use of health data for decisions and actions to improve the quality of health services and achieve performance goals is the vital ingredient of shared accountability [26]. Among possible organisational and behavioural determinants, decisions based on supervisor directives and managers seeking feedback were found to be determinant factors for data quality [27]. Even though there is existing knowledge of the poor quality of immunisation data and inconsistencies in reporting, to our knowledge, there is not enough comprehensive evidence on how to increase data use in order to improve data quality and enhance accountability.

In this research project, our hypothesis is that low data quality and inconsistent reporting might be due to the low level of data use at each level of the health system. If all those involved in the health system hierarchy use and evaluate the data, data quality might improve. Thus, our overall theory is that increased data use will increase data quality and, in turn, improve accountability in immunisation programme data management. We know from existing evidence that data quality is low, but we do not know the role of data use in evidence-based decision-making as a strategy to alleviate data quality issues not only in immunisation but also in other programmes.

To fill this knowledge gap, we will conduct an implementation science study to identify organisational, technical and individual level factors affecting data use, which in turn affects data quality. The main knowledge needed is the perception and level of use of data for decision-making, data users and producers perceptions, and practice about data use and the role of supervision and community leaders in increasing data use at the community level.

### Research question

How can the use of data within the immunisation programme be increased in order to improve data quality and ensure greater accountability?

### Objectives

1. To explore how immunisation data is reported and used for decision-making to improve immunisation services.
2. To assess the role of supervisory visits to increase data use, improve data quality and ensure accountability in immunisation programmes.
3. To explore interaction and feedback mechanisms within the health information system actors at district, facility and community level.
4. To explore existing community level engagement approaches that can be leveraged to increase data use, improve data quality and ensure accountability in immunisation programmes.

## Methods

### Study setting

The site for this study will be the North Gondar region, which is one of the largest rural areas among the nine zones in Amhara region, Northern Ethiopia. The North Gondar region has an estimated area of 48,204.39 km^2^ and an estimated population density of 60.23 people per km^2^. The region has 22 *woreda* (districts) and 557 *kebele*’s (small administrative units), and an estimated total population of 5,631,777, of which 1,656,251 are above 18 years of age. The region is one where health facilities are widely spread in a large geographic area. A quarter of the administrations are categorised as in hard-to-reach, remote settings according to the Ministry of Health categorisation of districts.

### Study design

The study design will be a mixed method investigative approach to gain an in-depth understanding of the current data reporting, supervision and feedback mechanisms in the immunisation programme and the underlying factors that affect data use to inform decisions, especially within local level decision-makers and community representatives. It is an implementation science study that will include health workers and community representatives at different levels of the health system, starting from the primary healthcare unit (comprised of one health centre and five health posts) to the regional health bureau level.

#### Quantitative part

Quantitative data will be obtained through document review of monthly, quarterly and annual reports as well as from other feedback documents to understand the immunisation data reporting methods and interaction channels available. Quantitative data will be collected from various sources, including facility, district and zonal reports as well as related feedback documents from the district to data producers. In this review, we will not focus on the quality of the data as there is already enough evidence on this; however, we will assess the type of feedback the facilities receive after reporting to the district. We will also focus our assessment on the existence of plans and reports, the frequency of supervisions and their feedback, feedback content, and any involvement of community representatives in the process.

#### Qualitative part

Qualitative data will be gathered through key informant interviews with semi-structured questions directed to district managers, facility-level data users, data producers and community representatives. The key informant interview tool for decision-makers, termed the ‘Key informant interview–Decision-makers’ has 25 questions, including questions about the level of use of information for decision-making, the challenges in using data for decision-making, and the organisational capacities to process and use data. The key informant interview tool for data producers, termed the ‘Key informant interview–Data producers’ also has 25 questions, including questions about the flow of information, the support mechanism (the quality of supervision visits, feedback and supplies), and the interaction between them and data users. The key informant interview tool for data users, termed the ‘Key informant interview–Data users’ has 32 questions, including questions about their role, the level of data use, and barriers in producing and using data. Community representatives, including *kebele* administrators and health development armies, will be the detailed focus of this study. The key informant interview tool for community representatives, termed the ‘Key informant interview–Community representatives’ has 33 questions, including questions about the level of involvement in health data decision-making, planning health programmes, and the best methods and options to make them involved in data use for the community and in decision-making.

### Data collection

#### Key informant interviews

A pre-test will be undertaken to validate our in-depth interview tool. The research team members will conduct one-on-one interviews with four groups of participants from districts, facilities and the community selected using purposive sampling. The interview will include 10 district managers, 15 facility-level data users and supervisors, 20 data producers, and 10 community representatives. The number of participants will be determined by the research team based on similar previous research experiences as required to reach information saturation level.

The data collection will take place from August 2017 to January 2018. Each of the key informant interviews will take approximately 1 hour and will be conducted by two people in the research team and recorded with respondents’ consent. There will be a designated note-taker to make sure we have summarised notes for immediate review.

Interviews will be conducted at locations and times that are convenient for the key stakeholders and that ensure their privacy and confidentiality. In the interviews, at least the research assistant and one of the Principal Investigators will be there. Interviews will be tape-recorded. The data collection will be performed in the local language (Amharic) and transcribed for analysis.

#### Document review

Document review at each level of the reporting hierarchy will be conducted on three sources, namely facility reports, district reports and any feedback documents from the district to data producers. Standard data collection formats will be used to collect quantitative data from the selected health facilities at each level. Since all facilities report to the district, all facility reports to the district from January 1 to December 30, 2017, will be reviewed.

All facility reports will be kept in the Health Management Information System unit of the district in both soft and hard copies. We will review the softcopy documents, but when cross checking is necessary, we will also use the hard copy records. Since this aggregate information does not have personal identifiers, no individual data will be collected in reviewing the documents. A data extraction sheet will be prepared to summarise how the report was compiled, if there was any supportive supervision in the process and whether any feedback was provided during report writing.

The extracted information will be first discussed with experts working in each institution to make sure they are comfortable with the information and whether they believe that the proposed reporting would make it possible for them to be identified by their bosses. If so, we will make the information is more generalised until the experts are comfortable with it, thus ensuring that document producers are not at risk.

In both data collection processes, we will focus on community level data users and producers to obtain the best evidence on how to make data use community driven and community owned in the future. We will also investigate the existing community level engagement approaches to leverage them for better data use in the future. The document review will take place from August, 2017 to January, 2018.

### Data analysis

Interview qualitative data transcripts and notes from the document review will be compiled and reviewed by at least two research team members for quality of evidence. Data from the interviews will be analysed according to theme using NVivo software. Qualitative thematic comparative analysis to provide in-depth information about clusters of constructs that contribute to success or failure of implementation will also be considered. The quantitative document review data (facility, district and feedback reports) will be entered in a computer database using Epidata ver. 3.0 (The EpiData Association) as the interface and later exported into SPSS version 20 (SPSS Inc.) for descriptive statistics. Frequencies and cross-tabulations will be computed between the different sources of data. We will also perform descriptive statistics for the attitude questions related to data use with a scale of five. A *P* value level of 0.05 will be considered as significant.

## Discussion

The value of health data and information is determined by its utilisation in decision-making. Data use is wrongly understood in the health system as data reporting, aggregating numbers and sending those to someone at higher level. We refer to health data use as the process whereby the health system, at different levels, analyses health-related data, interpreting and elaborating it for the better understanding of the situation, and synthesises the data for decision-making on actions for which concerned bodies will take responsibility and be held accountable for it based on evidence. Quality data provides accurate and timely information to manage services and aids to prioritise and ensure the best use of resources [28].

With regards to taking action, the health system is largely accountable to act and make services available and accessible to the community. Overall, the health system should be responsive to the health concerns of the community by utilising the available routine data for decision-making [21, 26]. In line with this, the need for organised, accessible, timely and accurate data for health decision-making is affected by various factors at different levels [27]. Therefore, this study will provide an in-depth understanding of individual, technical and organisational level factors that influence the routine utilisation of data at local levels and in turn its effect on the quality of immunisation data.

Appropriate and timely use of health and health-related information for decision-making is an essential element in the process of transforming the health sector. Decisions at different levels of the health sector can only be effective if they are backed with accurate and reliable information [13, 20]. Despite the intensive effort to improve the efficiency of information systems in the past few years, the utilisation of information at the local level remains a challenge in the Ethiopian health system. Verification and feedback systems also improve the quality of data and the effectiveness of local and hierarchical utilisation of information [12]. This study will inform the implementation targets set under one of the pillars of the health sector transformation plan that is the information revolution.

While there is plenty of evidence on data quality, there is a lack of sufficient data on the role of evidence-informed local decision-making in improving data quality [11]. Following on from this, the overarching goal of our proposed research is therefore to close this gap for routine decision-making activities. For this to be practical, identifying and acting on the barriers at different levels is critical.

A key hypothesis in this paper is that data quality and data use are interrelated, namely that poor quality data will not be used and, due to this lack of use, the data will remain of poor quality. Conversely, greater use of data will help to improve its quality, which will in turn lead to greater data use. Hence, our study will provide robust context-specific implementation science evidence to assist in moving the information revolution agenda forward [14].

Overall, this will be one of the first studies in Ethiopia to mine innovative approaches to be used to improve culture of evidence use. In addition, we will propose mechanisms to ensure shared accountability and community engagement for local health data use in decision-making, which in turn will improve data quality.

## Conclusion

The comprehensive evidence to be generated from this research will provide a holistic understanding of how evidence generated at the local level is being continuously used for decision-making and to identify the barriers and facilitators at different levels of the health system. We will also develop a guidance document on how local data can be used for local decision-making and shared accountability in the health system.

## Abbreviations

EPI: Expanded Program on Immunization
